# Supplementary material for: The causal role of circulating inflammatory markers in osteoporosis: a bidirectional Mendelian randomized study
Source: Front Immunol. 2024 Jul 18;15:1412298. doi: 10.3389/fimmu.2024.1412298 (PMC11291241; doi:10.3389/fimmu.2024.1412298)
Supplement: Supplementary file 1 [file DataSheet_1.zip › supplementary material/OSTEOPOROSIS-CIM/GCST90274827.tsv.gz_leave_one_out.pdf]

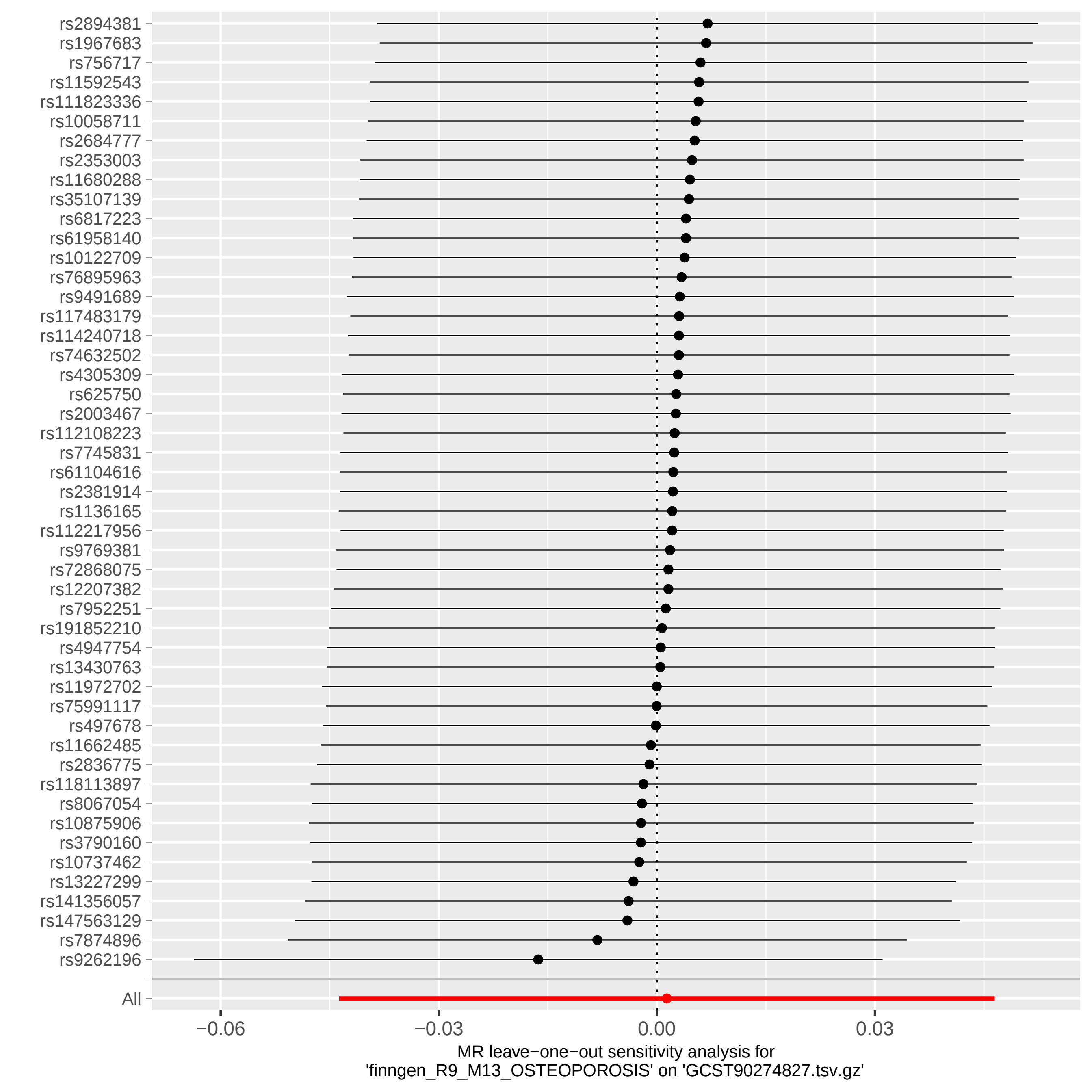
rs2894381
rs1967683
rs756717
rs11592543
rs111823336
rs10058711
rs2684777
rs2353003
rs11680288
rs35107139
rs6817223
rs61958140
rs10122709
rs76895963
rs9491689
rs117483179
rs114240718
rs74632502
rs4305309
rs625750
rs2003467
rs112108223
rs7745831
rs61104616
rs2381914
rs1136165
rs112217956
rs9769381
rs72868075
rs12207382
rs7952251
rs191852210
rs4947754
rs13430763
rs11972702
rs75991117
rs497678
rs11662485
rs2836775
rs118113897
rs8067054
rs10875906
rs3790160
rs10737462
rs13227299
rs141356057
rs147563129
rs7874896
rs9262196
All
−0.06
−0.03
0.00
0.03
MR leave−one−out sensitivity analysis for
'finngen_R9_M13_OSTEOPOROSIS' on 'GCST90274827.tsv.gz'
